# Popular Science Monthly

**Published:** 1878-06

**Authors:** 


THE POPULAR SCIENCE MONTHLY FOR JUNE.—The first article in *The Popular Science Monthly* for June is by Dr. F. L. Oswald, and is a vivid eulogy of the physical culture of the Greeks, under the title of "The Age of Gymnastics." Herbert Spencer continues his interesting papers on the "Evolution of Ceremonial Government, by treating the history, derivation, and significance, of "Obeisances." There is much curious information in the paper, and many interesting views, together with explanations in connection with this social custom. Dr. George M. Beard continues his important discussion of "The Scientific Study of Human Testimony," devoting himself now to the statement of the limitations of the senses, the limitations of the human brain in disease, the involuntary life and the necessity of a reconstruction of the principles of evidence. Under the title, "Scientific Courses of Study," Prof. F. W. Clarke contributes an able paper to the practical side of the subject of scientific education. "The Cardiff Giant and other Frauds" are discussed in a very entertaining way by Dr. A. G. Stockwell. "The Order of Nature," by C. S. Peirce, is the fifth in his series of "Illustrations of the Logic of Science." It is a bold and original statement, and will profoundly interest all thinkers who care about the more recent problems of the phenomena of Nature. The most important article in the number is that on "Brain-Forcing," by T. Clifford Allbutt, M. D. This paper is worth a year's subscription to the *Monthly* for every parent or teacher who has responsible charge of the young. Altogether the June number is very strong on the educational side. "The illustrated articles are "Water-Waves and Sound, Waves," by Lockyer; "The Gigantic Extinct Armadillos," and "The Pigmy Monkey." There are also a portrait and sketch of the late Professor Hartt.

